# The Impact of Serums Calcium 25-Hydroxy Vitamin D, Ferritin, Uric Acid, and Sleeping Disorders on Benign Paroxysmal Positional Vertigo Patients

**DOI:** 10.3390/audiolres14040054

**Published:** 2024-07-16

**Authors:** Abdulbari Bener, Ahmet Erdoğan, Ünsal Veli Üstündağ

**Affiliations:** 1 Department of Biostatistics and Public Health, School of Medicine, Istanbul Medipol University, Istanbul 34810, Turkey; 2 Department of Evidence for Population Health Unit, School of Epidemiology and Health Sciences, The University of Manchester, Manchester M13 9PR, UK; 3 Department of ENT, Medipol International School of Medicine, Istanbul Medipol University, Istanbul 34810, Turkey; 4 Department of Biochemistry, Medipol School of Medicine, Istanbul Medipol University, Istanbul 34810, Turkey

**Keywords:** benign paroxysmal positional vertigo, vitamin D, ferritin, calcium, uric acid, sleeping disorder

## Abstract

Objective: This study’s objective was to identify the factors and impact of serums calcium 25-Hydroxy vitamin D, ferritin, uric acid, and sleeping disorders on benign paroxysmal positional vertigo (BPPV) patients. Methods: This is a case and control design study. The consecutive patients’ visits (age, older than 25 years) with idiopathic BPPV were recruited in the present study. For each patient, 3:1 sex and age-matched healthy people were assigned as the control. The study comprised 177 patients with BPPV and 656 controls. The study included biochemical, clinical, physical examinations, PSQI sleep quality, supine roll test, and Dix–Hallpike test for the diagnosis of all patients, and pure-tone audiometry (PTA) was used to assess hearing. Univariate and multivariate stepwise regression analyses were used for statistical analysis. Results: The study comprised 833 patients with 295 males (35.4%) and 538 females (64.6%) who were between 25 and 70 years old. Of a total of 833 participants, 177 were BPPV patients, and 656 subject were normal. The results shown that there were significant differences between the BPPV and the normal group in terms of BMI (*p* = 0.039), physical activity (*p* = 0.003), cigarette smoking (*p* = 0.035), nargile-waterpipe use (*p* < 0.001), diabetes (*p* < 0.001), hypertension (*p* < 0.001), congestive heart failure (CHF) (*p* < 0.001), neurology (*p* < 0.001), tinnitus (*p* < 0.001), dizziness (*p* < 0.001), headache (*p* < 0.001), vitamin D (*p* = 0.004), calcium (*p* = 0.004), magnesium (*p* < 0.001), potassium (*p* = 0.019), phosphorus (*p* < 0.001), haemoglobin (*p* < 0.001), serum glucose (*p* < 0.001), HbA1c (*p* < 0.001), triglyceride (*p* < 0.001), systolic BP (*p* = 0.004), diastolic BP (*p* = 0.008), and microalbuminuria (*p* = 0.005); ATP III metabolic syndrome (*p* = 0.038), IDF metabolic syndrome (*p* = 0.034), and poor sleep (*p* = 0.033). In terms of the type of BPPV, the posterior canal was the most commonly affected (*n* = 126, 71.2%), followed by the horizontal (*n* = 43, 24.3%) and anterior canal (*n* = 8, 4.5%). The analysis indicated that serum ferritin (*p* < 0.001), uric acid (*p* < 0.001), blood pressure (*p* < 0.001), dizziness (*p* < 0.001), cigarette–water-pipe smokers (*p* = 0.004), headaches/migraines (*p* = 0.005), calcium (*p* = 0.007), vitamin D deficiency (*p* = 0.008), sleepiness (*p* = 0.016), physical activity (*p* = 0.022), CHF (*p* = 0.024), and tinnitus (*p* = 0.025) were considered as risk predictors for BPPV. Conclusions: The results revealed that the serum levels of vitamin D, ferritin, uric acid, and calcium are low among the study population and supplementation could be considered as prevention in BPPV patients.

## 1. Introduction

The symptoms of benign paroxysmal positional vertigo (BPPV) are typically triggered by movements of the head and are characterized by short-duration episodes with spontaneous remission. BPPV is a common peripheral vestibular disorder [1]. BPPV causes dizziness, nausea vomiting, and nystagmus. BPPV recurrences are common, with a yearly recurrence incidence of 15–20% [2,3,4]. BPPV is a disorder of the inner ear with a high rate of recurrence. Vascular disorders, migraine, and autoimmune disorders have been considered facilitating factors for relapsing episodes [2]. The incidence rate increases with age. BPPV is an important health problem worldwide and can occur at any age, with a higher incidence in the elderly [2,3,4,5]. BPPV patients, especially women, have sleep disturbance when compared to the normal group [4].

BPPV has a lifetime prevalence that is twice as high in females as in males [4]. The prevailing explanation for the pathology is that BPPV occurs due to the displacement of otoconia from the only utricle, leading to their adhesion to the cupula or migration into the semicircular canals. The otoconia metabolism and remodeling is a dynamic process that requires calcium and is regulated by vitamin D, which may involve the pathogenesis of BPPV [2,3,5]. Other factors, such as advanced age and estrogen fluctuation, have been cited as risk factors for BPPV [6]. Some studies have investigated the association of uric acid (UA) and vitamin D levels with BPPV and reached different conclusions [7,8]. Generally, between 40 and 60 percent of BPPV patients may exhibit a range of non-specific symptoms, including imbalance, dizziness, lightheadedness, anxiety, depression, and falls, particularly in older adults [8].

Studies have indicated that vitamin D, uric acids, and ferritin supplementation may prevent BPPV and reduce hearing problems [9,10,11,12,13]. Therefore, monitoring vitamin D levels may be necessary in BPPV patients to reduce the recurrence and severity of the disease [14,15]. Moreover, vitamin D supplementation indirectly reduces oxidative stress in BPPV patients [2,3,5,9,10].

Oxidative stress is the disruption of the balance between oxidants (reactive oxygen species, ROS) and antioxidants. While ROSs are necessary for normal cell function, excessive production can cause damage to cell components. Oxidative stress plays a role in sleep-deprivation-related disorders [7,8,12]. Uric acid is derived from purine metabolism and is the most abundant endogenous antioxidant in blood plasma. In addition, increased levels of free iron in serum contribute to increased oxidative stress through the Fenton reaction [7]. Carrier molecules in blood plasma, such as hemoglobin, ferritin, and albumin, act as antioxidants by binding to free iron due to their binding properties. Oxidative stress leads to impaired metabolic processes. Increased glycolysis due to increased oxidative stress plays a role in many diseases [2,3,9,11,12,13]. This study’s objective was to identify factors and impact of serum calcium 25-Hydroxy vitamin D, ferritin, uric acid, and sleeping disorders on benign paroxysmal positional vertigo (BPPV) patients.

## 2. Subjects and Methods

### 2.1. Participants

A cross-sectional study was conducted on participants aged 25 to 70 years who sought treatment at the ear, nose, and throat (ENT) clinics and outpatient clinics at Istanbul Medipol University Hospital between May 2017 and March 2021. The study was conducted in accordance with the Declaration of Helsinki (1964) and received ethical clearance and approval from the ethics committee (RP# 10840098-772.02.01-E.49746 and IRB# 10840098-604.01.01-E.3193).

Based on the previously reported prevalence of 20% [13], a sample size of 1110 subjects was calculated with a 99% confidence interval and 3% error of estimation. Of these, 833 agreed to participate in the study, resulting in a response rate of 75%.

The diagnostic criteria for benign BPPV were formulated by the Committee for the Classification of Vestibular Disorders of the Bárány Society [1]. Participants with a history of head trauma, vestibular neuritis, Meniere’s disease, migraines, sudden hearing loss, ear or otologic surgery, malignancy, autoimmune disorders, infectious diseases, malignant hepatic conditions, stroke, autoimmune diseases, and currently taking supplements were excluded.

The study included various demographic factors, such as age, gender, BMI, smoking and alcohol habits, physical activity, and existing health conditions. Central positional vertigo was evaluated through magnetic resonance angiography (MRA) and magnetic resonance imaging (MRI). The diagnosis of BPPV was confirmed through the patient’s physical examination, a medical history, and specialized testing such as the Dix–Hallpike or roll test. These tests were used to diagnose both BPPV patients and control group.

#### Biochemical and Vitamin D Assessment

The biochemical parameters measured in the study included hemoglobin, serum ferritin, magnesium, potassium, phosphorous, creatinine, fasting glucose levels, HbA1c, HDL, LDL, cholesterol, albumin, triglyceride, uric acid, bilirubin, triglyceride, uric acid, systolic and diastolic blood pressure, and microalbuminuria. The serum levels of vitamin D in the participants were measured using competitive radioimmunoassay (RIA) with the 25-hydroxy vitamin D test, which was conducted using the DiaSorin method, as described in previous studies [9,13,16].

### 2.2. Hearing Test

Pure-tone audiometry (PTA) was utilized to measure hearing sensitivity, classify the type and degree of hearing impairment, and assess the peripheral and central auditory systems (Garson Stadler GSI 61 and Inter-acoustics AC40 Clinical audiometer, Inter-acoustics, Assens, Denmark). The evaluation of hearing loss was described and classified as hearing loss exceeding 26 dB or as normal (≤26 dB) [13,16].

#### Pittsburgh Sleep Quality Index (PSQI)

The Pittsburgh Sleep Quality Index (PSQI) was determined by the method of Buysse et al. [17]. The total score of the PSQI was used to categorize the participants’ sleep quality as “good” (total score ≤ 5), “average” (total score between 6 and 8), or “poor” (total score ≥ 9).

### 2.3. Statistical Analysis

To evaluate the normality of the data, the one-sample Kolmogorov–Smirnov test was used. In order to determine significant differences between the two groups, the independent Student’s *t*-test was used for normally distributed data, while the Mann–Whitney U test was used for non-normally distributed data. Categorical variables were compared using the chi-square (χ^2^) test. Pearson’s correlation was utilized for continuous variables. Multivariate stepwise linear regression analysis was conducted to identify the risk factors associated with BPPV. All p-values were two-tailed and a p-value of less than 0.05 was considered statistically significant.

## 3. Results

This cross-sectional study comprised 833 patients, with 295 males (35.4%) and 538 females (64.6%) who were between 25 and 70 years old. Of a total of 833 participants, 177 were BPPV patients, and 656 were in the control group. Table 1 presents the clinical characteristics and socio-demographics of the BPPV patients compared to the control subjects. The results indicated that there were significant differences between the two groups in terms of several parameters and physical activity (*p* = 0.003), including BMI (*p* = 0.039), cigarette smoking (*p* = 0.0359), nargileh-waterpipe use (*p* < 0.001), diabetes (*p* < 0.001), hypertension (*p* < 0.001), CHF (*p* < 0.001), neurology (*p* < 0.001), tinnitus (*p* < 0.001), dizziness (*p* < 0.001), and headache (*p* < 0.001).

Table 2 shows the clinical biochemistry variables of the benign paroxysmal positional vertigo patients compared to the control group. Significant statistical differences were observed between patients with BPPV and normal subjects with regard to the value of serum vitamin D (*p* = 0.004), serum Ferritin (*p* < 0.001), hemoglobin (*p* < 0.001), magnesium (*p* < 0.001), calcium (*p* = 0.004), potassium (*p* = 0.019), phosphorus (*p* < 0.001), fasting glucose (*p* < 0.001), HbA1c (*p* < 0.001), triglyceride (*p* < 0.001), systolic BP (*p* = 0.004), diastolic BP (*p* = 0.008), and micro-albuminuria (*p* = 0.005), the level of vitamin D (*p* = 0.007), ATPIII metabolic syndrome (*p* = 0.038), IDF metabolic syndrome (*p* = 0.034), and PSQI sleep quality (*p* = 0.033). Further, the BPPV characteristics are posterior canal (*n* = 126, 71.2%), horizontal (*n* = 43, 24.3%), and anterior canal (*n* = 8, 4.5%).

Table 3 shows the correlation coefficients (Pearson’s r) between various selected parameters and benign paroxysmal positional vertigo. As can be seen from this table, most of the clinical biochemistry parameters appeared to be statistically significant with BPPV.

Table 4 shows the multivariate regression analysis for the predictors of BPPV. The analysis shows that serum Ferritin (*p* < 0.001), uric acid (*p* < 0.001), blood pressure (*p* < 0.001), dizziness (*p* < 0.001), cigarette–water-pipe smokers (*p* = 0.004) headaches/migraines (*p* = 0.005), calcium (*p* = 0.007) vitamin D deficiency (*p* = 0.008), sleepiness (*p* = 0.016), physical activity (*p* = 0.022), congestive heart failure (CHF) (*p* = 0.024), and tinnitus (*p* = 0.025), were considered as risk predictors for BPPV. 

## 4. Discussion

Our findings indicate that there is a high prevalence of vitamin D deficiency among Turkish individuals with BPPV, which is in line with previous studies conducted in the United Kingdom, USA, Iran, Iraq, and China, where low serum vitamin D levels have been observed [13,18,19,20,21]. 

The present study shows that there were lower levels of 25(OH) D in BPPV patients compared to normal subjects. Also, the multivariate regression analysis showed that vitamin D, uric acid, and calcium deficiency were associated with and predictors of BPPV. Further, in the current study, it has been observed that vitamin D deficiency among BPPV patients was stronger, with a twice as high risk in women than in men. A recent study shows that vitamin D intake can lower the recurrence of BPPV [5,7,10,13]. This is confirmative with the current study.

The higher incidence with advancing age is associated with decreased calcium and vitamin D levels and thinning and weakening of the bones. But, serum 25(OH)D levels might be affected by several parameters, such as gender, age, climate, hormone, geographic area, nutrition, lifestyle habits, and sleeping disturbance [4,13,18,19,20,21]. Most recently, a meta-analysis reported that vitamin D deficiency was a significant indicator among patients with recurrent episodes of BPPV [22,23,24]. The present study demonstrated a strong positive relationship between obesity, being overweight, and BPPV and hearing loss [3,10,11,12,13,15]. In this study, in addition to the decreased vitamin D levels, a significant decrease in calcium and phosphorus levels was observed. Studies show that vitamin D deficiency can affect ear and balance functions. Therefore, BPPV patients with vitamin D deficiency may need to be followed up regularly and take vitamin D supplements [3,10,11,12,13,15].

Overall, it has been reported that, as vitamin D levels increased, tinnitus and vertigo problems decreased [9,10,11,12,13]. Also, it has been recommended by studies [3,4] that it would be beneficial to pay attention to this and to increase vitamin D levels through dietary adjustment. Therefore, vitamin D supplementation can be highly effective and beneficial in the treatment and prevention of BPPV [13,18,19,20,21,22,23,24]. 

Several studies have shown that sleeping disorders are related to vertigo and tinnitus impairments [9,13]. Recently, a study was performed and explored the association between BPPV and sleep disorders. Through the analysis, they have found that patients with BPPV have sleeping disturbances and significantly decreased sleep quality [25,26,27]. In the current study, BPPV revealed significantly lower sleep quality and efficiency compared to the normal group. Also, a more recent study reported that poor sleep quality (PSQI score > 5) was found in 35% of patients in the BPPV group and 13.3% in the normal group [4]. Those results are confirmative with the current study. 

In addition to the decrease in antioxidant levels in BPPV patients, it has been reported that there is a decrease in serum uric acid and albumin levels [7,12]. Uric acid is an endogenous antioxidant. In addition, transporter molecules, such as hemoglobin, serum ferritin, and albumin in blood plasma, act as antioxidants due to their free-iron-binding properties. In our study, in addition to a decrease in uric acid and ferritin levels, there was a significant decrease in hemoglobin levels compared to the normal group. Yuan et al. [25] conducted a study on a Chinese population and found low serum hemoglobin A1C and albumin levels to be associated with a high prevalence of BPPV, but it is still unclear whether these factors are linked to BPPV recurrence. Yamanaka et al. [28] reported a positive correlation between vertigo disease and aging in metabolic syndrome patients. In addition, increased HbA1c levels were observed in our study, in addition to the increased fasting glucose level in the BPPV group. Moreover, BPPV patients showed a higher prevalence of ATP III metabolic syndrome, IDF metabolic syndrome (*p* = 0.034), and poor sleep quality compared to the normal group. Further studies are needed to elucidate the mechanism underlying oxidative stress-induced glycosylation in BPPV patients.

The present study has some limitations. First, because the data collection was so time-consuming, some of the participants did not want their test. So, they were excluded from the study. Second, the information on smoking was collected by self-report, which is subject to various method biases. Third, the assessment of comorbid disease status may be misclassified or may not be accurate. Lastly, fourth, the data concerning sleepiness obtained from the scale were subjective and may not be very precise.

## 5. Conclusions

Mostly, women and the older age group are affected by BPPV disease. The current study results indicated that the serum levels of vitamin D, ferritin, uric acid, and calcium are low among the study population and supplementation could be considered as a preventative in BPPV patients. Additionally, poor sleep quality was highly significantly more common among BPPV patients compared to the normal group. 

## Figures and Tables

**Table 1:** The socio-demographic and clinical characteristics of benign paroxysmal positional vertigo patients compared to control subjects (N = 833).

	BPP Vertigo Hearing Loss ≥26 dB *n* = 177	Control <26 dB *n* = 656	*p*-Value
Age groups (in years):			
<50	69 (39.0)	220 (33.5)	
50–59	53 (29.9)	228 (34.8)	0.338
>60	55 (31.1)	208(31.7)	
Gender:			
Male	63 (35.6)	232 (354)	1.000
Female	114 (64.4)	424 (64.6)	
BMI (kg/m^2^):			
Normal (<25 kg/m^2^)	67 (39.9)	196 (29.9)	
Overweight (29–30 kg/m^2^)	57 (32.2)	277 (42.2)	0.039
Obese (>30 kg/m^2^)	53 (29.9)	183 (27.9)	
Physical activity:			
Yes	64 (36.2)	164 (25.0)	0.003
No	113 (63.8)	492 (75.0)	
Cigarette Smoking:			
Yes	34 (19.2)	85 (13.0)	0.035
No	143 (80.8)	571 (87.0)	
Nargileh-Waterpipe smoke:			
Yes	31 (17.5)	57 (8.7)	0.001
No	146 (82.5)	599 (91.3)	
Family history of diabetes:			
Yes	39 (21.5)	74 (11.3)	0.001
No	138 (78.5)	582 (88.7)	
Family history of hypertension:			
Yes	44 (24.9)	87 (13.3)	0.001
No	133 (75.1)	569 (86.7)	
Congestive Heart Failure (CHF):			
Yes	53 (29.9)	82 (12.5)	0.001
No	124 (70.1)	574 (87.5)	
Family history Neurological disease:			
Yes	51 (28.8)	93 (14.2)	0.001
No	126 (71.2)	563 (85.8)	
Tinnitus:			
Yes	50 (32.1)	108 (16.5)	0.001
No	127 (67.9)	548 (83.5)	
Dizziness:			
Yes	65 (36.71)	117 (17.8)	0.001
No	112 (82.3)	539 (82.2)	
Headache/migraine:			
Yes	60 (33.9)	95 (14.5)	0.001
No	117 (66.1)	501 (85.5)	
Characteristics of BPPV:			
Posterior	126 (71.2)		
Horizontal	43 (243)		
Anterior	8 (4.5)		

**Table 2:** Clinical description of biochemistry variables in benign paroxysmal positional vertigo patients compared to control subjects (N = 833).

	BPP Vertigo ≥26 dB Mean ± SD	Control <26 dB Mean ± SD	*p* Value
Serum Vitamin D (ng/mL)	19.04 ± 8.37	21.19 ± 9.00	0.004
Serum Ferritin (ng/mL)	144.42 ± 51.70	180.29 ± 52.01	0.001
Haemoglobin (g/dL)	12.96 ± 1.50	13.50 ± 1.90	0.001
Magnesium (mmol/L)	0.66 ± 0.09	0.95 ± 0.10	0.002
Potassium (mmol/L)	3.46 ± 0.56	3.49 ± 0.53	0.680
Calcium (mmol/L)	1.87 ± 0.90	2.00 ± 0.91	0.005
Phosphorous (mmol/L)	1.53 ± 0.71	1.74 ± 0.67	0.001
Creatinine (mmol/L)	74.46 ± 15.56	74.75 ± 14.42	0.815
Fasting Glucose (mmol/L)	6.70 ± 1.19	6.37 ± 1.00	0.001
HbA1c (%)	6.98 ± 1.31	6.28 ± 0.87	0.001
Cholesterol (mmol/L)	4.78 ± 098	4.75 ± 0.80	0.685
HDL (mmol/L)	1.05 ± 0.31	1.10 ± 0.29	0.064
LDL (mmol/L)	1.80 ± 0.71	1.87 ± 1.00	0.3269
Albumin (mmol/L)	41.92 ± 4.05	42.25 ± 5.353	0.437
Billirubin (mmol/L)	7.07 ± 2.47	7.34 ± 2.54	0.210
Triglyceride (mmol/L)	1.66 ± 0.72	1.49 ± 0.34	0.001
Uric Acid (mmol/L)	254.0 ± 60.48	281.3 ± 62.39	0.001
Systolic Blood Pressure (mm Hg)	131.90 ± 10.47	129.91 ± 8.28	0.005
Diastolic Blood Pressure (mm Hg)	81.02 ± 8.20	79.16 ± 8.35	0.009
Microalbuminuria	12.20 ± 3.81	7.68 ± 1.86	0.348
	*n* (%)	*n* (%)	
Vitamin D Level			
Deficiency 25(OH)D <20 ng/mL	107 (60.5)	310 (47.3)	
Insufficiency 25(OH)D 20–29 ng/mL	41 (23.2)	210 (32.0)	0.007
Optimal 25(OH)D 30–80 ng/mL	29 (16.4)	136 (20.7)	
ATP III Metabolic Syndrome			
Yes	37 (20.9)	95 (14.5)	0.038
No	140 (79.1)	561 (85.5)	
IDF Metabolic Syndrome			
Yes	46 (26.0)	123 (18.8)	0.034
No	131 (74.0)	533 (81.2)	
Sleep quality:			
Good sleep (PSQI score < 5)	63 (34.3)	293 (41.8)	
Average sleep (PSQI score 6–8)	49 (26.6)	205 (29.2)	0.026
Poor sleep (PSQI score > 8)	72 (39.1)	203 (29.0)	

**Table 3:** The Pearson correlation coefficient between benign paroxysmal positional vertigo and selected parameters (N = 833).

Parameters	Benign Paroxysmal Positional Vertigo Correlation Coefficient r	*p*-Value Significance
Serum Vitamin D (ng/mL)	0.099	0.004
Serum Ferritin (ng/mL)	0.273	0.001
Calcium (mmol/L)	0.100	0.004
Uric acid (mmol/L)	0.171	0.001
Phosphorous (mmol/L)	0.128	0.001
Systolic Blood Pressure (mm Hg) Hg)	−0.099	0.004
Diastolic Blood Pressure(mm Hg)	−0.092	0.008
Cigarette smoking	0.073	0.035
Nargileh	0.117	0.001
ATP Metabolic Syndrome	0.072	0.038
IDF Metabolic Syndrome	0.074	0.034
Tinnitus	0.123	0.001
Dizziness	0.187	0.001
Fatigue	0.158	0.001
Headache/migraine:	0.204	0.001
Nausea	0.160	0.001

**Table 4:** The predictors of benign paroxysmal positional vertigo using multivariate stepwise regression analysis (N = 833).

Independent Variables	Unstandardized Coefficients		Standardized Coefficients Beta	*t* Test	Significance *p* Value
	B	Standard Error			
Serum Ferritin	0.197	0.025	0.273	8.169	0.786
Uric acid	0.462	0.112	0.151	4.614	<0.001
High blood pressure	0.135	0.033	0.119	3.475	<0.001
Dizziness (Yes)	0.176	0.054	0.177	3.278	<0.001
Cigarette–Water-pipe smokers	0.132	0.046	0.099	2.860	0.004
Headache/migraine	0.109	0.039	0.103	2.812	0.005
Calcium	0.295	0.109	0.089	2.715	0.007
Vitamin D deficiency	0.314	0.122	0.090	2.692	0.008
Sleep disturbance (Yes)	0.122	0.050	0.083	2.421	0.016
Physical activity	0.066	0.030	0.071	2.335	0.022
Congestive Heart Failure (CHF)	0.096	0.043	0.087	2.267	0.024
Tinnitus (Yes)	0.129	0.057	0.124	2.252	0.025

## Data Availability

Data available on request from the correspondence authors.
